# Fat embolism syndrome in a patient that sustained a femoral neck fracture: A case report

**DOI:** 10.3389/fmed.2022.1058824

**Published:** 2022-12-08

**Authors:** L. A. S. den Otter, B. Vermin, M. Goeijenbier

**Affiliations:** ^1^Department of Intensive Care Medicine, Spaarne Gasthuis, Haarlem, Netherlands; ^2^Department of Intensive Care Medicine, Erasmus Medical Center, Rotterdam, Netherlands

**Keywords:** fat embolisms, fat embolism syndrome, cerebral fat embolisms, “Starfield” pattern, patent foramen ovale (PFO)

## Abstract

**Background:**

We present a case of a patient with a femoral neck fracture that shows neurological impairment and respiratory distress 1 day after trauma, caused by the Fat Embolism Syndrome with the presence of Cerebral Fat Embolisms.

**Case summary:**

A 75 year old female remained unresponsive after a hemi arthroplasty was performed because of a 1 day old femoral neck fracture. She rapidly developed respiratory insufficiency and an obstructive shock with right ventricle dilatation on transthoracic echocardiography. The diffusion-weighted MRI brain images showed the “Starfield” pattern, a radiologic phenomenon typical for FES. During 3 weeks of ICU admission the neurologic state slowly ameliorated.

**Conclusion:**

The rare FES is a clinical diagnosis with mainly respiratory, neurologic and dermatologic symptoms in the setting of a trauma patient. Fat embolisms are able to reach the brain without the presence of a patent foramen ovale to cause neurological symptoms. Diagnosing FES remains challenging but the distinctive “Starfield” pattern on MRI scans is promising.

## Introduction

Fat embolisms (FE) are defined as the presence of fat particles in the blood circulation and Fat Embolism Syndrome (FES) is a rare condition that occurs when FE result in symptoms and/or organ failure. FE seem to be common after trauma, with prospective studies and autopsy reports showing high incidences of FE (41–94%). Only a few of these patients however develop FES (recent reported incidence ranges from 1–11%) and even less patients are diagnosed with CFE (1–6). The pathophysiology of FES remains speculative and diagnosing FES challenging. Our case report illustrates the unpredictable clinical course of FES and the challenges when diagnosing FES.

## Case report

A 75 year old female presented to the emergency room with a dislocated femoral neck fracture after a fall off her electrical bike on the left hip. She had a history of chronic obstructive pulmonary disease gold (COPD)III, hypertension with stage III renal failure and an abdominal aortic aneurysm, for which an endovascular aortic repair was performed (‘16) and later on an aortic bifurcation graft was inserted (‘18). Since it was an isolated fall on her hip and the neurological exam was normal, a computed tomography (CT) scan of the brain was not performed. She was admitted to the surgical ward with systemic opioids and a femoral nerve catheter with bupivacaine 0.25% as analgesia and surgical fixation by a hemi arthroplasty was performed the next day. Anesthesia consisted of a spinal block with bupivacaine 0.5% and midazolam (2mg) was administered as part of sedation. After surgery she remained unresponsive with a Glasgow Coma Scale (GSC) of 4 (E2M1V1) and on examination the patient appeared to be sweating and showed an impaired breathing pattern. Her vital signs showed a mild tachycardia (110/min) with normal blood pressure. Arterial blood gas analysis showed a mild hypercapnia (pCO2 7.2 kPa). Other important lab values contained a hemoglobin level of 7.1 mmol/l, a leukocyte count of 26.4 × 10^9^/l, a C-reactive protein level of 103 mg/l and a blood glucose level of 6.5 mmol/l. Naloxone was administered as an antidote for the opioids and Flumazenil as an antidote for midazolam, both without clinical effect. Fysostigmine was administered to treat a possible central anticholinergic syndrome, also without any clinical effect.

The patient was admitted to the Intensive Care Unit (ICU) with non-invasive ventilation for further diagnostics and treatment. The neurologist was consulted to exclude neurologic pathology for the impaired consciousness. Neurologic examination showed an impaired consciousness with a GCS of 4 (E2M1V1), no signs of lateralisation, pupils normal in size, shape and responsive to light, normotone muscle state and symmetrical hyporeflexia with indifferent Babinski reflexes. Within 1 hour her consciousness improved to a GCS of 7 (E2M4V1). A CT brain was not performed because of an improvement in consciousness and vital signs that were not typical for a neurologic origin of an impaired consciousness.

**Figure 1:**
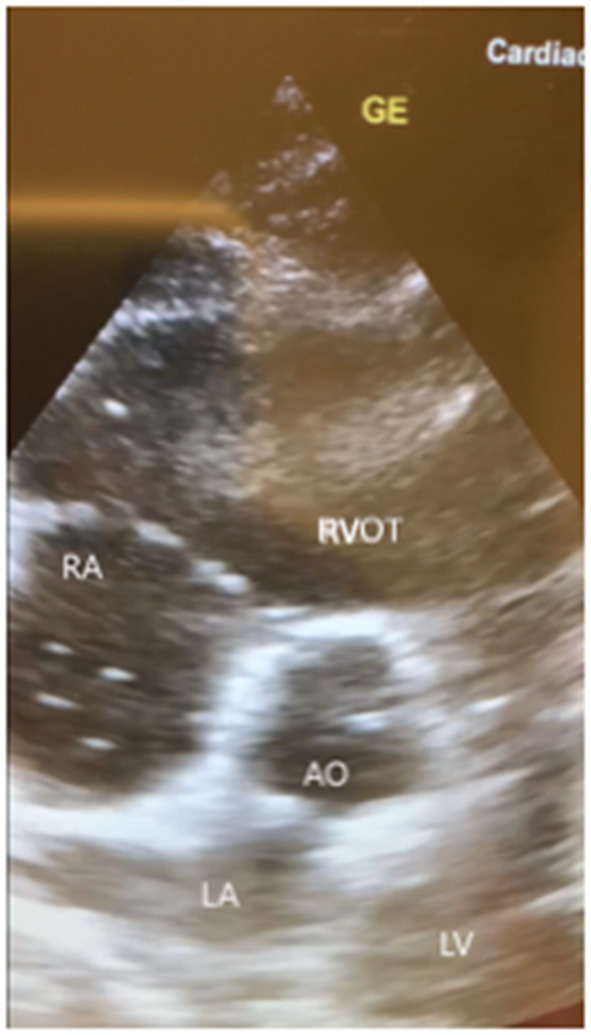
Images of bedside transthoracic echocardiography (subcostal 5-chamber view, short axis) of our patient showing a remarkable dilated right ventricle with multiple hyperechoic floating dots. RA, right atrium; RVOT, right ventricle outflow tract; LA, left atrium; LV, left ventricle; AO, aorta; GE, ultrasound marker.

A few hours later, her clinical status deteriorated with an acute drop in blood pressure and hypoxia which made us decide to intubate and start mechanical ventilation. Subsequently a central venous line for vasopressors was placed. A chest X-ray was performed which showed no abnormalities. Bedside transthoracic echocardiography (TTE) of the heart showed a remarkable dilatation of the right ventricle with multiple hyperechoic particles (Figure 1). This made us think of Fat Embolism Syndrome (FES) as a cause for her neurologic, respiratory and hemodynamic symptoms. Her electrocardiogram (ECG) showed a sinus tachycardia without signs of acute ischemia.

A CT of the brain without contrast was performed and showed no abnormalities. A CT with contrast (arterial phase) of the thorax was performed which excluded pulmonary embolisms, but did confirm a widened right ventricle. On the third day of admission both a transthoracic and transesophageal ultrasound of the heart were performed which showed a dilatation of the right ventricle, no signs of ischemia and moreover no patent foramen ovale (PFO) or other intracardiac shunt. Having excluded other more common causes for the symptoms, we suspected FES as cause for the obstructive shock. Furthermore, we suspected cerebral fat embolisms (CFE) as a possible cause for the impaired consciousness. As recommended, we continued with “best supportive care”, which mainly included mechanical ventilation for respiratory insufficiency and inotropic and vasopressor medication for an obstructive shock.

**Figure 2:**
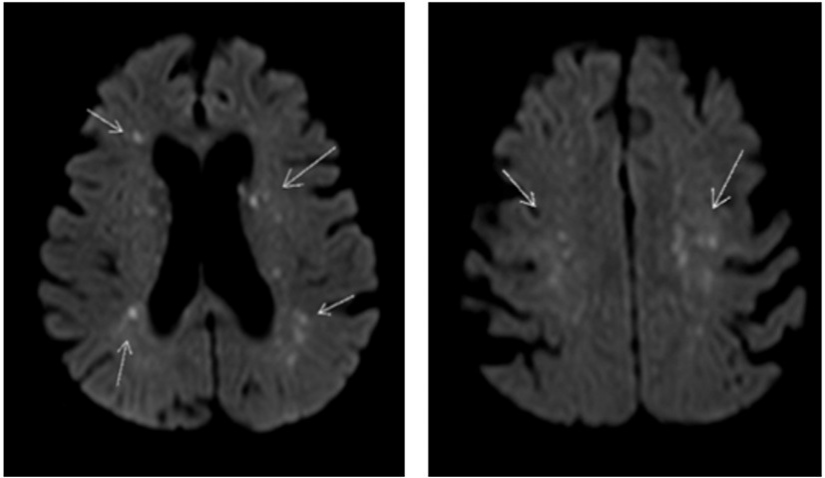
Diffusion weighted MRI scan of the brain of our patient showing multiple small foci of reduced diffusion and edema. Arrow shows small foci of reduced diffusion and edema.

On day 4 of admission a CT scan of the brain with venous contrast was performed to exclude a basilar artery occlusion and did not show any abnormalities. On day 6 a magnetic resonance imaging (MRI) scan of the brain was performed which showed diffuse small foci of reduced diffusion and edema on the diffusion-weighted scan (Figure 2) as well as petechial hemorrhage on the T2-weighted scan. These lesions are described in literature as the “Starfield” pattern, a radiographic phenomenon suggestive for CFE. Patient remained hemodynamically stable after recovery and with the use of a tracheostoma she was weaned of mechanical ventilation. In light of her COPD gold III, we chose rapid weaning of mechanical ventilation using early tracheostomy to minimize muscle loss. During 3 weeks of ICU admission the neurologic state slowly ameliorated to a maximal EMV score with a partial recovery of verbal communication and an improving critical illness polyneuropathy.

## Discussion

FES has mainly been reported to develop after traumatic fractures and sometimes after other traumatic events like burns and soft tissue injury (7). In rare cases, FES has been described after non-traumatic events like pancreatitis, sickle cell disease, corticosteroid therapy and lipid infusions (8). Risk factors for the development of FES include long-bone (mainly femur- and tibial shaft) and pelvic fractures, the presence of bilateral/multiple fractures and late fixation of the fractures (9). FES has been diagnosed in the adult and pediatric population and is mostly seen in males under the age of 30 years, probably because of the high incidence of trauma in this age group.

Symptoms of FES typically appear 24 to 48 h after sustaining the fracture and predominantly include a triad of respiratory, neurological and dermatological symptoms. Respiratory symptoms like tachypnea and dyspnea are the most common and respiratory signs can include hypoxemia, pulmonary edema and acute respiratory distress syndrome (ARDS). Although the severity of respiratory distress varies, fulminant respiratory failure which requires mechanical ventilation has been reported in 44% of FES cases (10). Massive FE can even lead to obstructive shock and can progress to acute right heart failure (11). Neurological features are reported in >50% of cases and can include confusion, an altered consciousness, seizures and/or focal neurological deficits (1). Again, the extent of these neurological impairment varies from mild confusion to refractory status epilepticus and brain death. Next to respiratory and neurologic symptoms and -signs, a petechial rash is the third most reported sign of FES that is mainly seen at the axillae, trunk and sclera. In our case, symptoms developed approximately 26–28 h after trauma and consisted of neurological impairment and respiratory failure. We did not observe a petechial rash.

The pathophysiology behind FES remains unknown but several theories have been proposed to clarify its pathogenesis. The mechanical theory suggests that bone marrow derived fat droplets enter the venous circulation as a result of increased medullary pressure caused by the fracture. These will at some point lead to occlusion, presumably in the pulmonary arterial circulation (7, 12). Activation of the clotting cascade by thromboplastin may play a role in this process, since FE in lung tissue appear to be surrounded by platelet aggregations. This “coagulation theory” will lead to expansion of the fat droplets in size, making it more likely to occlude a vessel (13). These mechanisms however do not account for the delay between the traumatic events and the symptoms.

The biochemical theory states that tissue lipases, products of a systemic inflammatory response induced by a stress state after trauma, break down fat particles into free fatty acids. These free fatty acids are toxic for and injure the endothelium, making it more susceptible for obstruction. This two-hit theory can explain the delay between the traumatic event and symptoms of FES and can also account for the non-traumatic cases of FES (14, 15).

FE can enter the arterial circulation by an intra-cardiac shunt like a PFO or through the lung capillary bed as micro-emboli to disseminate in the systemic (micro)circulation (16, 17). The latter being the suspected route to the brain in our patient since she did not seem to have a PFO.

Diagnosing FES is challenging since at this moment, there is no diagnostic tool that can diagnose FES. Diagnosis is often established at autopsy when FE are shown in organ systems which correlates with premortal clinical symptoms fitting for FES. Since FES is a rare disorder, more common causes of the respiratory or neurological symptoms must be excluded. Laboratory results and radiologic tests are all non-specific for FES. The most common lab findings include anemia (or a drop in hemoglobin) and thrombocytopenia.

A X ray or CT scan of the chest can show no abnormalities like in our case report or show non-specifics abnormalities like bilateral diffuse infiltrates and pulmonary oedema (18). Transoesophageal and transthoracic ultrasound of the heart have been used to detect fat emboli (16, 19). Our ultrasound images (Figure 1) are very similar to the images shown in case reports (20, 21).

A CT scan of the brain will mainly be used to exclude more common causes of neurological symptoms like cerebral strokes and like our case, usually shows no abnormalities in FES patients. Remarkable is a typical pattern seen on the diffusion weighted MRI images of brains of patients with CFE. This so-called “Starfield” pattern consists of bilateral widely spread hyper intense foci of restricted diffusion and edema in both white and gray matter and at this point seems to be the most distinctive feature to FES (22–24). This “Starfield” pattern is easily recognized on diffusion weighted MRI images of our patient. Petechial hemorrhages can be seen in subacute stages op T2 MRI images, which was also seen on MRI images our patient (25). Repeating the MRI scan has shown complete resolution of these lesions in patients that clinically recovered from FES, suggesting MRI images correlate with the extent of neurological impairment (26, 27). Unfortunately, we do not have a repeated MRI scan of the brain.

The lack of a diagnostic tool makes FES a clinical diagnosis. Although no diagnostic scoring system has been validated, the most commonly used diagnostic criteria include the Gurd and Wilson criteria, consisting of major and minor criteria. Major criteria contain the triad of respiratory distress, cerebral symptoms (without the presence of head injury) and a petechial rash. Minor criteria include tachycardia, a fever, renal or retinal involvement, jaundice and lab results (thrombocytopenia, a descending hemoglobin level, an elevated erythrocyte sedimentation rate and fat macroglobulinemia). Diagnosis requires either two major criteria or one major and four minor criteria (28). The patient in our case met two major criteria (respiratory and cerebral involvement) and at least three minor criteria (tachycardia, thrombocytopenia, a drop in hemoglobin level) and according to Gurd and Wilson's criteria would suggest the presence of FES.

Treatment options are limited and at this moment. Heparin has been proposed as a therapeutic agent because it stimulates lipase enzyme activity which lowers intravascular lipid concentrations. Since the FES mainly occurs in trauma patients, the concomitant risk of bleeding makes the use of heparin controversial. With the biochemical theory in mind, corticosteroids were thought to be beneficial in treating and even preventing FES. Conflicting results of studies combined with a high rate of infectious complications are the main reason corticosteroids are not recommended as standard treatment for FES (29).

Due to the lack of therapeutic options, best supportive care to optimize oxygenation, ventilation and circulation is the only recommended treatment. Literature states a mortality rate with best supportive care lower than 10% and a full recovery of symptoms in most patients within days to 1 week (30). In a smaller subset of patients, FES sets a fulminant course and leads to death, with the main causes being respiratory failure, shock, and/or braindead.

Preventative measures have not been thoroughly researched yet and mainly focus on timing of surgery and surgical techniques. Early fixation of fractures has been suggested to reduce the incidence of FES by retrospective and prospective studies (31–33). For instance, using techniques to lower the intramedullary pressure during surgery (for instance using reamer systems with vacuum or venting options or applying intraoperative medullary cavity suction and/or irrigation) reduces the presence of FE (visualized by intraoperative TEE) (34, 35).

## Conclusion

Whereas the presence of FE is common, FES is a rare disorder. Knowledge of the typical triad of respiratory, neurologic and dermatologic symptoms in the setting of a trauma patient is important to recognize FES. Diagnosing FES remains a challenge since there is no validated laboratory test or imaging modality that can diagnose FES. It remains a diagnosis of exclusion but distinctive patterns on MRI scans are promising and have potential to become the gold standard in diagnosing FES. Echocardiography should be used as a tool since it is easy to perform, non-invasive and might help raise suspicion for FES, like in our case. It is important to realize that even without the presence of a PFO, FE are able to reach the brain and cause neurological symptoms. Due to a lack of treatment options, best supportive care is the current therapeutic management with relatively good prognostic outcomes. However, in a minority of patients a fulminant FES is observed with potentially fatal outcomes. Patience is key in treating FES, as our case illustrates. This case report illustrates the turbulent clinical course and the remarkable recovery potential of FES and emphasizes the importance of a high suspicion of FES in trauma patients.

## Data availability statement

The original contributions presented in the study are included in the article/supplementary material, further inquiries can be directed to the corresponding author/s.

## Author contributions

LO study of literature and writing first draft. BV and MG reviewing and editing the first draft. All authors listed issued final approval of the version that is submitted.
